# The effect of intraoperative lung protective ventilation vs conventional ventilation, on postoperative pulmonary complications after cardiopulmonary bypass

**DOI:** 10.15171/jcvtr.2017.38

**Published:** 2017-12-15

**Authors:** Mohammad Mahdi Zamani, Atabak Najafi, Saloomeh Sehat, Zinat Janforooz, Pooya Derakhshan, Faranak Rokhtabnak, Mehrdad Mesbah Kiaee, Alireza Kholdebarin, Masoud Ghorbanlo, Mohammad Hossein Hemadi, Mohammad Reza Ghodraty

**Affiliations:** ^1^Department of Anesthesiology and Pain Medicine, Firoozgar Hospital, Iran University of Medical Sciences, Tehran, Iran; ^2^Department of Anesthesiology and Critical Care, Tehran University of Medical Sciences, Tehran, Iran; ^3^Department of Anesthesiology and Pain Medicine, Moheb Hospital, Iran University of Medical Sciences, Tehran, Iran; ^4^Department of Anesthesiology and Pain Medicine, Rasoul Akram Hospital, Iran University of Medical Sciences, Tehran, Iran

**Keywords:** Coronary Artery Bypass Grafting, Ventilator-Induced Lung Injury, Mechanical Ventilation, Recruitment Maneuver, Lung Protective Strategy

## Abstract

***Introduction:*** This study aimed to evaluate the effects of high positive-end expiratory pressure (PEEP) and low tidal volume (TV) and recruitment maneuver, on postoperative pulmonary complications (PPCs) after coronary artery bypass grafting (CABG) surgery.

***Methods:*** This study is a randomized double blind clinical trial on 64 patients who were undergoing CABG surgery, and were randomly divided into two groups of conventional ventilation (C-Vent) with TV of 9 mL/kg and PEEP=0 cm H2O, and lung protective ventilation (P-Vent), with 6 mL/kg TV and PEEP=10 cm H2O with recruitment maneuver every 30 minutes. Measures of PPCs and modified clinical pulmonary infection score (mCPIS), were assessed for the first 24 hours of postoperative time in order to evaluate the pulmonary complications.

***Results:*** P-Vent with 31 patients and C-Vent with 30 patients, participated in the stage of data analysis. Demographic, and preoperative laboratory results showed no significant difference between two groups. During surgery, cardiovascular complications were higher in P-Vent group (*P *= 0.61) but pulmonary complications were higher in C-Vent group (*P *= 0.26). Extubation time was not significantly different between two groups, and also components of arterial blood gases (ABG) of 24 hours after surgery showed no significant difference between the two groups. Pathologic changes in the chest X-ray (CXR) of 24 hours after surgery, were lower in P-Vent group, but the difference was not significant (*P *= 0.22). The PPC criteria was less positive in P-Vent (2 patients) vs 9 patients in C-Vent group (*P *= 0.02) and mCPIS score was significantly lower in P-Vent group (1.2 ± 1.4) than C-Vent group (2 ± 1.6) (*P * = 0.048).

***Conclusion:*** Lung protective strategy during and after cardiac surgery, reduces the postoperative mCPIS in patients undergoing open heart surgery for CABG.

## Introduction

In recent years, the use of low tidal volume (TV) (protective ventilation), on healthy lungs has been studied and it has represented beneficial effects compared to conventional ventilation.^1^ Review articles are generally in agreement about the beneficial effects of protective ventilation in acute respiratory distress syndrome (ARDS) and lung injury in intensive care units (ICUs),^2,3^ and in recent studies, researchers are exploring the effects of different components of ventilations during anesthesia, to reduce lung injury by ventilation in patients with normal respiratory system during surgery.^4^

Theory of multiple trauma (multiple hit theory) was reported as the main mechanism of ventilator-induced lung injury (VILI) in healthy lungs. In VILI the “hit” is intensified in healthy lung by mechanical ventilation even if the least harmful setting was set.^5^ Lung protective ventilation and open lung ventilation can reduce VILI^6^ with several components of protective ventilation strategy, such as low TV,^7^ higher positive-end expiratory pressure (PEEP),^8^ and recruitment maneuver.^9^

The coronary artery bypass grafting (CABG) surgery under cardiopulmonary bypass (CPB) is often associated with high postoperative organ dysfunction, such as renal, intestinal, and lung complications. Lellouche et al reported the correlation between high TV and more postoperative organ dysfunction, in patients undergoing cardiac surgery.^10^ Lung complications that are more correlated with anesthesiologists management in ICU, were consisted of pulmonary atelectasis and weak Expansion, acute lung injury and postoperative pneumonia. These complications increase patient hospitalization time and ICU admission.

The objective of this investigation was to compare high PEEP and low TV ventilation and intermittent recruitment maneuver with conventional ventilation on postoperative pulmonary complications (PPCs) of CABG under CPB.

## Materials and Methods

This study is a double blind randomized controlled trial on patients undergoing CABG surgery under CPB, who were referred to a tertiary hospital with the approval of ethics committee of Iran University of Medical Sciences. We also registered the study in the Iranian Registry of Clinical Trials website (IRCT) (subset of WHO International Clinical Trials Registry Platform [ICTRP]) identifier: IRCT2015040115774N3; http://www.irct.ir/). All patients signed the informed consent.

Inclusion criteria were body mass index (BMI) less than 30 kg/m^2^, age over 18 years and less than 70 years and nonsmokers (nonsmoker or cigarette cessation for 8 weeks before surgery and ≤10 packet per year) entered our study. Exclusion criteria were emergency surgery, valve surgery, past history of thoracic surgery, chronic obstructive pulmonary disease (COPD), systolic heart failure (ejection fraction <40%), restrictive pulmonary diseases and asthma history, pregnancy, history of sleep disorders, repeated systemic corticosteroid treatment (inhaler or oral), liver or neuromuscular disorders, alcohol and drug abuse, anesthetic drug allergy, treatment with immunosuppressive drugs for chemotherapy or radiation therapy in the last 2 months, hemoglobin level below 10 mg/dl, albumin level less than 3 g/dL, continuous hemodynamic instability, resistant shock and non-early extubation (extubation after 6 hours from end of surgery)‏.

Demographic data and time of weekly exercise were recorded. Then chest X-ray (CXR), echocardiography, pulmonary function test (PFT), complete blood count (CBC) and creatinine were checked. Preoperative sspirin 80 (mg/d) and Atorvastatin 20 (mg/d) were continued in all patients. Angiotensin II receptor antagonist and Angiotensin converting enzyme inhibitors were discontinued 24 hours before surgery in all patients.

Sixty-four patients were enrolled into the study and using block Randomization with STATA software were divided into two groups of 32 patients, protective ventilation (PV group) and conventional ventilation (CV group). The sample size calculation formula was: n = [(Zα/2 + Zβ)2 × 2(standard deviation)2/ (µ1-µ2)2] where n = sample size required in each group, μ1 = mean of Modified Clinical Pulmonary Infection Score (mCPIS**)** in protective ventilation group, μ2 = mean of mCPIS in conventional ventilation group, μ1-μ2 = clinically significant difference, Zα/2: 5% level of significance (1.96), Zβ: 95% power (1.96) and standard deviation = 1.195. We designed a pilot study was performed among 10 patients (5 in each group) in which μ1 was measured as 1.05 and μ2 as 1.25. Therefore, n was calculated as 30 for each group which gave us a total sample size of 60.

A left radial arterial line and a 14 or 16 Gauge venous line in addition to the routine venous line of the ward were placed under infiltrative anesthesia. Then serum therapy was started (8-10 mL/kg of normal saline) and standard monitoring was connected.

All patients received 0.05-0.1 mg/kg morphine sulfate intramuscular, 30 minutes before transferring to the operating room. They also were pre-oxygenated with fraction of inspired oxygen (FiO2) = 1 for 3 minutes before induction of anesthesia. Fentanyl 3-10 μg/kg and etomidate 0.15-0.3 mg/kg and then 0.15-0.2 mg cisatracurium were prescribed. After 3 minutes, patients were intubated with single-lumen PVC tracheal tube (ID: 7 for women and ID: 7.5 for men) and tube cuff pressure was adjusted between 20 to 25 cm H₂O. Ninety seconds before intubation, 1.5 mg/kg intravenous lidocaine was used.^11^

A right internal jugular central venous catheter was placed and IV infusion of 0.25-0.5 μg/kg/min midazolam and (continuous) fentanyl at 0.1-0.03 μg/kg/min started and cisatracurium 0.03 mg/kg was administered every 30 minutes for the maintenance of anesthesia; in addition, isoflurane (0.6-1.5%) in 100% oxygen and poropofol (25-100 μg/kg/min) were administered during CPB based on patients BiSpecteral index (BIS) which was being kept between 40-60.

Intraoperative monitoring included invasive and noninvasive blood pressure, pulse oximetry, end tidal Co2 (ETCo2), temperature control, urine output and heartbeat measurement. The following data were recorded before induction, after induction of anesthesia, and also once before extubation: arterial blood gases (ABG), blood pressure, peripheral blood oxygen saturation and ETCo2. During the operation, the following data were recorded: volumes of crystalloids, colloids and any blood products, blood loss and urine output.

All Patients received 1500 mL of priming solution containing normal saline, NaHCO3 7.5% (45 mEq), 20% mannitol (5 mL/kg) and heparin. Heparin (primary bolus 3 mg/kg) was administered before the establishment of CPB. After inducing the anticoagulation with heparin, activated clotting time (ACT) was kept over 480 seconds. CPB was established with a membrane oxygenator (Terumo System 1^TM^, Terumo, Leuven, Belgium) with target flow rates of 2.4 to 2.8 L/min/m^2^ for all patients. Leukocyte-depleted packed red blood cells (PRBCs) were given when hemoglobin was <7 g/dL. Moderate hypothermia to 32°C and cold (4-8°C) cardioplegia concentrations were the same (K+ 20 mmol/L for arrest induction and 10 mmol/L for maintenance) in all patients. Furthermore, α-stat acid-base gas managing was used, and the goal range for PaO2 was 200-300 mm Hg. During CPB, norepinephrine or nitroglycerine were used to maintain arterial pressure between 60-80 mm Hg. Heparin was reversed with protamine sulfate (1 mg/1 mg of heparin). Before weaning from CPB, all patients were rewarmed to 36°C and all the work on the protocol for weaning from CPB was done. Ventricular flutter or fibrillation was treated immediately with defibrillation. Internal paddles are applied directly to the heart to deliver 10 to 20 J of electricity. If ventricular arrhythmias persist or recur, an antiarrhythmic drug, usually lidocaine or amiodarone, was infused. There was no difference in surgical technique between patients.

### Mechanical Ventilation

Mechanical ventilation was performed by Drager-Fabius anesthesia machine and the volume control mode (V-CMV) with FiO2 = 0.4, I/E ratio of 1:2 was set. Respiratory rate was adjusted to maintain normocapnia (starting with respiratory rate of 12 breaths/min) and ETCo2 between 30-35 mm Hg.

Ventilation protocol was TV of 6 mL/kg based on predicted body weight (PBW), and PEEP of 10 cm H₂O, and intermittent recruitment maneuver every 30 minutes, in PV group. Recruitment Maneuver was done after induction of anesthesia, then every 30 minutes, concomitant to relaxant injection, at the end of surgery, and before weaning from mechanical ventilator, in stable hemodynamic status and in the presence of a physician.

In the CV group, ventilation protocol was TV of 9 mL/kg based on PBW, and PEEP = 0 cm H₂O(ZEEP), without recruitment maneuver.

At the on-pump time, patients of both groups had no ventilation and no PEEP.

During surgery, complications were recorded and classified in two groups of cardiac complications and lung complications as follow:

1. Pulmonary complications: peripheral oxygen saturation less than 90% and / or end tidal carbon dioxide greater than 45 mm Hg for more than 1 minute, necessary to change the settings for TV and / or respiratory rate
2. Cardiac complications: any kind of arrhythmia, systolic blood pressure over 150 mm Hg or less than 90 mm Hg, requiring vasoactive drugs.

All postoperative complications including pneumothorax, atelectasis, cardiac tamponade and re-operation were recorded.

### Recruitment maneuver

Recruitment maneuver was performed after induction of anesthesia, every 30 minutes during surgery and before weaning of mechanical ventilator just before extubation, as follows: for 3 breaths in a deep inhalation so that the peak pressure reached to 30 cm H₂O. In the event of a sudden blood pressure drop (MAP of less than 60 mm Hg) the maneuver would be carried out.

### ABG sampling

Analysis of ABG was recorded before intubation and after 24 hours from the intubation time, while the patient was in sitting position, and was breathing in room air. If the patient was using an oxygen mask, the mask was removed for 15 minutes, and if peripheral oxygen saturation reduced to less than 88%, maneuver was immediately stopped and ABG was taken and O2 was started for the patient.

### Transfer to ICU

After the surgery, patients were transferred to the ICU, and connected to the ventilator station with synchronized intermittent mandatory ventilation (SIMV) mode. Ventilator setting was PEEP = 5 cmH2O, TV of 6 mL/kg in PV group and respiratory rate was adjusted to maintain normocapnia between 30-35 mm Hg (starting with 12 breaths/min). CV group received PEEP = 3 cm H₂O and TV of 9 mL/kg.

For postoperative pain, all patients received morphine sulfate 0.05-0.1 mg/kg every 6 hours and in case of any delirious state, midazolam 1-2 mg up to 5 mg was added, in both groups. All patients received antibiotic prophylaxis and anti-thrombotic therapy in postoperative period.

Patients were extubated while they were awake and obeyed the commands, with no need to use any inotrope, breathing in continuous positive airway pressure (CPAP of 3-5 cm H₂O) with FIO2 <0.4, respiratory rate less than 20 breaths/min, PaO2 over 60 mm Hg, PaCO2 less than 45 mm Hg and the chest tube drainage reached to 50 mL/h. All patients were extubated by the same anesthesiologist. Early extubation was described as extubation time less than 6 hours (from the end of surgery).

After extubation, all patients received chest physiotherapy, cough stimulation techniques and incentive spirometry in both groups.

CXR and ABG were performed and PPC and mCPIS questionnaires were filled exactly after 24 hours from the intubation time.

### Pathologic findings in chest radiography

CXR was performed before and after 24 hours for all the patients in bedside. A radiologist blinded to the group of patients evaluated radiographs and considered these four items as pathologic features: increase thickness of interstitium, disventilated areas including minimal density change, atelectasis, and pleural effusion.

### Postoperative pulmonary complications

After 24 hours from intubation time, PPC was evaluated. The patients were evaluated for cough, increased mucus, chest pain, dyspnea, body temperature above 38°C and heart rate above 100/min. The answers were recorded as positive or negative. Positive PPC was defined as more than 3 positive scores of six (4, 5, or 6 of 6).

### Modified Clinical Pulmonary Infection Score

After 24 hours from intubation time, mCPIS was calculated based on adding score of following items, temperature, serum leukocyte count, tracheal tube discharge, the PaO2/FiO2 ratio and pathological changes in CXR.

### Statistical analysis

Statistical analysis was performed using SPSS version 19. Quantitative data were expressed as mean and standard deviation and qualitative data were expressed as frequencies and percentages. Quantitative variables were compared between two groups using Student’s *t* test. The analysis of qualitative variables was done by chi-square test between two groups. In all cases, *P*<0.05 was considered statistically significant.

## Results

**Figure 1:**
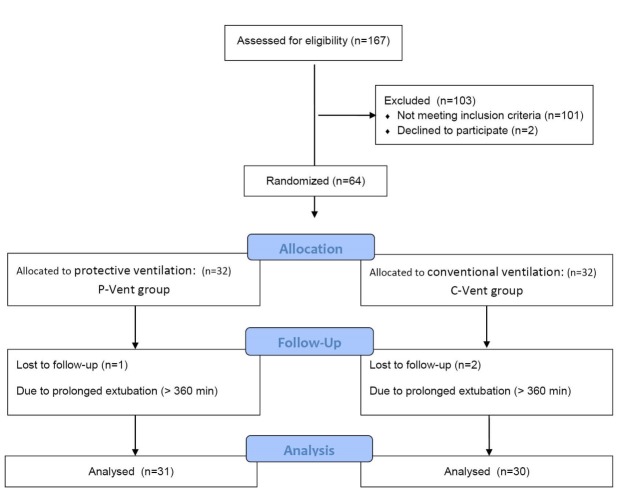
Consort Flow Chart.

In this study, a patient was excluded from PV group due to prolonged extubation time (640 minutes) because of heavy chest tube drainage. Also, two patients from CV group were excluded due to tachypnea (44/min) and metabolic acidosis leading prolonged extubation (respectively 520 and 670 minutes). Finally, 31 patients in the PV group and 30 patients in CV group were analyzed (Figure 1).

Demographic characteristics and blood test data for creatinine, hemoglobin and albumin the day before surgery did differ between two groups (Table 1). Moreover, characteristics of preoperative CXR and PFT, and ABG levels before induction of anesthesia were not different (Table 1).

**Table 1:** Demographic characteristic, clinical and laboratory data of patients before surgery in two groups (mean ±SD)

**Demographic Data**	**PV group** **(n =31)**	**CV group ** **(n = 30)**	***P*** ** value**
Age (y)	56 ± 9	57 ± 8	0.96
Sex (M/F)	14/17	15/15	0.34
BMI (kg/m^2^)	26.0 ±1.51	25.47 ± 1.39	0.15
Cigarette ≤10 PY (Y/N)*	15/16	14/16	0.31
ASA score (II/ III)	20/11	22/8	0.20
Exercise (min/wk)	58 ± 36	44 ± 48	0.67
History of TS (n)	0	0	-
Paraclinical Data			
EF (%)	43 ± 18	41 ± 19	0.52
FEV1 (l)	2.31± 0.48	2.20 ± 0.49	0.24
FEV1, % predicted	74.91 ± 8.99	76.59 ± 8.58	0.44
FVC (l)	2.51 ± 0.65	2.44 ± 0.73	0.15
FVC, % predicted	60.38 ± 13.62	67.30 ± 19.89	0.11
Laboratory data			
Hb (g/dL)	11.42 ± 2.70	12.10 ± 1.91	0.52
Albumin (g/dL)	4.21 ± 0.40	3.96 ± 0.71	0.26
Creatinine (mg/dL)	1.23 ± 0.28	1.16 ± 0.17	0.08
ABG			
pH	7.40 ± 0.02	7.42 ± 0.01	0.25
PCO2	37 ± 3	38 ± 2	0.57
PO2	121 ± 93	150 ± 85	0.17
HCO3	22.80 ± 2.82	22.45 ± 3.20	0.64
BE	-1.53 ± 0.33	-1.40 ± 0.94	0.25

ASA: American Society of Anesthesiologists; BMI: body mass index; Cigarette ≤10 PY: cigarette smoking less or equal 10 Pack Year; PV: Protective ventilation; CV: Conventional ventilation; M: male; F: female; TS: thoracic surgery; EF: ejection fraction; FEV1: forced expiratory volume in one second; FVC: forced vital capacity; Hb: hemoglobin; CXR: chest X-ray; ABG: arterial blood gas; PCO2: partial pressure of carbon dioxide; PO2: partial pressure of oxygen; HCO3: Bicarbonate; BE: base excess.

Intraoperative variables including operation time, the amount of blood loss and received packed red blood cell and other blood products, isotonic serum intake, urine output, and on-pump time, did not differ between two groups (Table 2). Intraoperative cardiac complications were more frequent in PV group (58% vs 33% in CV group) but lung complications were less in PV group (6% vs 17% in CV group) (Table 2). Intraoperative cardiac complications of PV group included 14 cases of hypotension, two cases of premature ventricular contraction (PVC) and two cases of hypertension. In CV group, there were eight cases of hypotension, one case of PVC and one case of ventricular tachycardia after pump which was made into sinus rhythm with 10 J, DC-shock (*P* = 0.06). Intraoperative lung complications of CV group included one case of low saturation (known as cardiac ischemia and cardiogenic pulmonary edema with pink bubbles and increased pulmonary artery pressure (PAP = 60 cm H₂O) in echocardiography) and two cases of increased ETCO2. In PV group, there was a case of low saturation and a case of increased ETCO2 (with pH = 7.1 who got DC-shock).

**Table 2:** Operation time, the amount of blood loss, the amount of received PC, the amount of received serum, urine output, on-pump time, and intraoperative complications of patients before surgery in two groups (mean ± SD)

**Characteristics**	**PV Group (n =31)**	**CV Group (n = 30)**	***P*** ** value**
Duration of surgery (min)	176 ± 42	182 ± 19	0.86
Isotonic serum infusion (mL)	2969 ± 812	3234 ± 732	0.17
IO Blood loss (mL)	325 ± 126	466 ± 230	0.33
PC transfusion (mL)	1.75 ± 0.72	1.50 ± 0.62	0.14
Other colloid* (n)	2	1	0.49
Urine output (mL)	1092 ± 343	1020 ± 290	0.36
On-pump time (min)	39 ± 11	37 ± 10	0.41
Cardiac complications (Y/N)	18/13 (58%)	10/20 (33%)	0.61
Lung complications (Y/N)	2/29 (6%)	5/25 (17%)	0.26

PV: protective ventilation; CV: conventional ventilation; IO bleeding: intraoperative bleeding; PC: packed cell (500 mL) *Patients who received other colloids moreover PC.

There were no significant differences between two groups in postoperative morphine and midazolam, extubation time and ABG of 24 hours after surgery (Table 3).

**Table 3:** The amount of received morphine and midazolam, extubation time, and analysis of CXR and ABG of 24 hours after surgery in two groups (mean ± SD)

**Data**	**PV group** **(n =31)**	**CV group** **(n = 30)**	**P value**
MS (g)	6.67 ± 2.22	7.0 ± 2.30	0.73
Midazolam (mg)	5.09 ± 1.45	5.46 ± 1.71	0.57
Extubation time (min)	305 ± 49	294 ± 43	0.53
ABG			
pH	7.36 ± 0.03	7.35 ± 0.3	0.55
PCO2	35 ± 5	36 ± 4	0.19
PaO2	142 ±59	134 ± 54	0.93
HCO3	22.05 ± 2.20	21.12 ±2.06	0.09
BE	-2.59 ± 2.96	-2.60 ± 2.0	0.97
Pathologic findings in CXR (Y/N)	22/9	26/4	0. 23

ABG: arterial blood gas; PV: protective ventilation; CV: conventional ventilation; CXR: chest x-ray; PCO2: partial pressure of carbon dioxide; PaO2: partial pressure of oxygen; HCO3: bicarbonate; BE: base excess; MS: morphine sulfate. *P < 0.05 is significant.

In the PV group, pathological findings in the 24 hours post-surgery CXR, was less than CV group (71% vs 87%), but the difference was not significant (*P* = 0.23) (Table 3).

The amount of positive PPC was lower in PV group (two patients in PV vs 9 patients in CV group) (*P* = 0.02) (Table 4) and mCPIS was significantly lower in PV group (1.2 ± 1.4) in comparison with CV group (2 ± 1.6) (*P* = 0.048) (Table 5).

**Table 4:** The results of PPC, 24 hours after surgery in two groups

**Components**	**CV group** **(n =30)**	**PV group (n = 31)**	***P*** ** value**
Cough, No. (%)	14 (47)	4 (13)	0.05*
Increased secretions, No. (%)	13 (43)	5 (16)	0.07
Dyspnea, No. (%)	18 (60)	6 (19)	0.001*
Chest pain, No. (%)	21 (70)	5 (16)	0.001*
Temperature >38°C, No. (%)	7 (23)	4 (13)	0.57
HR >100 beats/min, No. (%)	10 (33)	4 (13)	0.039*
PPC score (+/-)	9/21	2/29	0.02*

HR: heart rate; PPC: postoperative pulmonary complications; PV: protective ventilation; CV: conventional ventilation. *P < 0.05 is significant.

**Table 5:** The results of mCPIS, 24 hours after surgery in two groups

**Components**	**CV Group** **(n =30)**	**PV Group** **(n = 31)**	**P value**
Temperature, °C, n(%)			
≥36.1 and ≤38.4	25 (83)	28 (90)	0.73
≥38.5 and ≤38.9	3(10)	2(7)	
≥39 or ≤36	2(7)	1(3)	
Serum leukocyte, No. (%)			
≥4000 and ≤11.000	29 (97)	29(93)	0.55
<4000 or >11.000	1(3)	2 (7)	
<4000 or >11.000 and ≥50 band cell	0 (0)	0 (0)	
Tracheal discharge, No. (%)			
Few	19 (64)	20 (65)	0.70
Moderate	4 (13)	6 (19)	
Large	4 (13)	4 (13)	
Purulent	3 (10)	1 (3)	
Pao2/Fio2, mm Hg, No. (%)			
>240 or absence of ARDS	24 (80)	28 (90)	0.28
≤240 and presence of ARDS	6 (20)	3 (10)	
Chest x-ray, No. (%)			
No infiltrate	9 (30)	15 (48)	0.03*
Patchy or diffuse infiltrate	12 (40)	12 (39)	
Localized infiltrate	9 (30)	4 (13)	
mCPIS, mean ± SD	1.2 ± 1.4	2 ± 1.6	0.048*

PaO2: partial pressure of oxygen; FiO2: fraction of inspired oxygen; PV: protective ventilation; CV: conventional ventilation; mCPIS: Modified Clinical Pulmonary Infection Score. *P < 0.05 is significant.

Pneumothorax and atelectasis was not observed in any patient. Tamponade occurred in one patient which was evacuated by tapping under echocardiography guide. Second surgery was not performed during hospitalization in any of our patients.

## Discussion

We showed that intra and postoperative protective ventilation along with high PEEP and recruitment maneuver can be effective in reducing mCPIS and PPC in patients undergoing open heart surgery for CABG surgery.

Previous studies on lung protective ventilation in CABG surgery under CPB, practiced protective ventilation strategy only at the end of operation until extubation and reported protective effects on pulmonary complications.^12,13^ Some studies have only evaluated protective ventilation and recruitment maneuver in intraoperative period^14^ and several other studies have just investigated postoperative protective strategy.

In a study by Chaney et al,^13^ the effect of postoperative ventilation with respiratory rate (RR) of 16/min and TV of 6 cc/kg and PEEP = 5 was compared with conventional ventilation (RR = 8/min and TV of 12 cc/kg and PEEP = 5) in the ICU. In this study, three pulmonary complications including increase in airway pressure, decreased pulmonary compliance and increased pulmonary shunt were defined and assessed in 12 patients in PV group and 13 patients in CV group. They reported that Lung protective ventilation strategy reduced common lung injuries and improved pulmonary function after CABG surgery. Reduction in dynamic compliance was lower in protective ventilation group and it was also significantly lower about static compliance. Pulmonary shunt increased significantly in conventional ventilation group after surgery, but it did not increase in protective ventilation group. In the study of Chaney et al, lung protective ventilation was applied only after surgery while in our study it was used in both intra and postoperative period.

Similarly, Wrigge et al^12^ investigated ventilation effects only after the CABG surgery and among lung protective ventilation strategies, only TV differed between two groups (6 cc/kg vs 12 cc/kg). Finally, this study did not report any clinical changes but demonstrated significant reduction of systemic and pulmonary inflammatory cytokines in PV group in comparison with CV group.

In a large study in United States,^10^ a database of 3434 patients undergoing cardiac surgery was studied prospectively; patients were classified in three groups after admission to the ICU: TV below 10 cc/kg, conventional TV between 10-12 cc/kg and TV above 12 cc/kg. Then, the effects of TV were investigated on the outcomes of prolonged intubation in ICU, long term ICU stay, hemodynamic stability, and renal failure. It was reported that TV above 10 cc/kg is associated with organ failure (lungs and kidneys) and increased prolonged staying in ICU after heart surgery. High TV was significantly associated with long-term hospitalization and increasing mortality. Additionally, in this study, BMI above 30 kg.m-^2^ was reported as an independent risk factor for lung injury. The average proportion of TV to the patient’s real weight was 9.2 cc/kg in low TV (<10) group.

In 2014, a review article surveyed strategies affecting pulmonary complications particularly lung protective ventilation and noninvasive ventilation after surgery and after extubation in ICU in patients undergoing cardiac surgery. They concluded that among components of lung protective ventilation strategy and VILI prevention methods after cardiac surgery, only low TV ventilation efficacy was confirmed. It was considered as a factor in reducing lung injury and mortality in patients with healthy and unhealthy lungs. Other components such as PEEP and recruitment maneuver efficacy remained in controversy.^4^ Recently other ventilation strategies were investigated, but had little effects, such as adaptive support ventilation (ASV),^15^ but among these several strategies, noninvasive ventilation (NIV) have had positive results to decrease PPCs.^16^

To the best of our knowledge, our study is the first to investigate protective ventilation strategies with two TV under 10 cc/kg (9 cc/kg vs 6 cc/kg) and high PEEP (10 cm H₂O) and recruitment maneuver to reduce PPCs in patients undergoing cardiac surgery. We selected mCPIS and PPC scoring to calculate PPCs.

Severgnini et al studied 56 patients in two groups: protective ventilation (TV = 7 cc/kg of PBW, PEEP = 10 cmH2O and recruitment maneuver every 30 min) and conventional ventilation (TV = 9 cc/kg, ZEEP without recruitment maneuver) in patients undergoing abdominal surgery. PFT and CXR before and after surgery, PPC score and mCPIS scoring were used to evaluate pulmonary infections and other lung complications.^17^ In present study, PFT was performed only before the operation due to the lack of patients’ cooperation after open heart surgery to rule out obstructive and restrictive pulmonary disease. In the study of Severgnini et al, CXR pathological changes were reported to be significantly lower (*P* = 0.024), and arterial oxygenation was higher (*P* < 0.05) in the first day after surgery in protective ventilation group.

In our study on patients undergoing CABG, CXR pathological findings were less frequent in PV group but the difference was not significant with CV group (*P* = 0.23). Arterial oxygenation in PV group was higher but the difference was not significant (*P* = 0.93). Severgnini et al considered mCPIS as the main outcome of study and it was reported that mCPIS was reduced significantly by protective ventilation, which is in line with our findings (*P* = 0.048).

In our study, a PEEP = 5 cm H₂O was used in PV group after surgery in the ICU, because according to Hansen et al. PEEP = 8 is not superior to PEEP = 5 and may be associated with complications.^9^ Therefore, we suggested to replace PEEP = 10 during operation with PEEP = 5 in the ICU.

The aim of this randomized controlled trial was to evaluate the effect of lung protective ventilation on clinical pulmonary complications parameters, during surgery and postoperative until the extubation. This study did not aim to investigate the severe lung complications after surgery. As mentioned in the recent review article, pulmonary complications after heart surgery are common but serious severe pulmonary complications were reported to be infrequent.^4^

We evaluated the following items: (1) Peripheral arterial oxygenation, (2) Dyspnea, coughing and discharge (PPC), (3) Pathologic CXR findings such as minor density changes, small areas without ventilation, atelectasis and pleural effusion, and (4) mCPIS.

In this study, we eliminated confounder effects of BMI and age on pulmonary complications. In previous studies, pulmonary stress outcome between ventilation and no ventilation during CPB has been different^18^; therefore, we performed similar ventilation strategy (no ventilation no PEEP) during CPB and eliminated this confounder factor too.

In the present study, using high levels of PEEP in PV group was not associated with severe hemodynamic instability or receiving more packed red blood cell and other blood products. Also, recruitment maneuver in PV group did not cause hemodynamic instability or any life-threatening event such as decrease in systolic blood pressure and heart rate and other complications.

## Conclusion

In conclusion, our findings demonstrate that protective ventilation methods during and after heart surgery, with a low TV with intraoperative PEEP = 10 and postoperative PEEP = 5 combined with-recruitment maneuver during surgery can significantly reduce mCPIS score, after surgery and prevent PPC score in patients undergoing open heart surgery for CABG. However, long-term evaluations after surgery are recommended for future studies as a limitation in this study.

## Ethical approval

The ethics committee of Iran University of Medical Sciences approved the study.

## Competing interests

All authors declare no competing financial interests exist.
